# Estimation of the Duration of Antihypertensive Prescriptions: Validation of a Data‐Driven Approach Using Rotterdam Study Data

**DOI:** 10.1002/pds.70164

**Published:** 2025-06-02

**Authors:** Chau L. B. Ho, David Youens, Walter P. Abhayaratna, Max K. Bulsara, Jeff Hughes, Rachael Moorin, Sallie‐Anne Pearson, David B. Preen, Christopher M. Reid, Rikje Ruiter, Christobel M. Saunders, Bruno H. Stricker, John Stubbs, Frank J. A. van Rooij, Cameron Wright, Ninh Thi Ha

**Affiliations:** ^1^ School of Population Health, Faculty of Health Sciences Curtin University Perth Australia; ^2^ Cardiovascular Epidemiology Research Centre, School of Population and Global Health The University of Western Australia Perth Australia; ^3^ Canberra Health Services, ACT Canberra Australia; ^4^ School of Medicine and Psychology Australian National University Canberra Australia; ^5^ Institute for Health Research The University of Notre Dame Australia Fremantle Australia; ^6^ Curtin Medical School Curtin University Perth Australia; ^7^ PainChek Ltd Sydney Australia; ^8^ School of Population and Global Health The University of Western Australia Perth Australia; ^9^ School of Population Health University of New South Wales Sydney Australia; ^10^ The NHMRC Medicines Intelligence Centre of Research Excellence Sydney Australia; ^11^ Department of Epidemiology Erasmus MC – University Medical Centre Rotterdam the Netherlands; ^12^ Department of Internal Medicine Maasstad Hospital Rotterdam the Netherlands; ^13^ Medical School The University of Western Australia Perth Australia; ^14^ Melbourne Medical School The University of Melbourne Melbourne Australia; ^15^ The Royal Melbourne Hospital Melbourne Australia; ^16^ Independent Cancer Consumer Advisor Sydney Australia; ^17^ Fiona Stanley Hospital Murdoch Australia; ^18^ School of Medicine University of Tasmania Hobart Australia

**Keywords:** hypertension, pharmacoepidemiology, prescription duration, waiting time distribution

## Abstract

**Objectives:**

Administrative medicine dispensing data often omit prescribed duration, which is important for research on adherence or other pharmacoepidemiological topics. While the reverse waiting time distribution (rWTD) method has been widely used to estimate prescribed durations, its accuracy in real‐world dispensing data is unknown. We assessed the performance of the rWTD method against the actual prescribed duration recorded in the Rotterdam Study.

**Methods:**

100 725 antihypertensive (AHT) prescriptions from 2018 to 2019 were extracted from the Rotterdam Study's medicine data. Data were constructed into five scenarios with increasing variability in the number of medicines included and variations in prescribed duration. The rWTD with 10 random index dates with or without adjustment for the quantity of dispensed medicine was conducted in all scenarios. Relative differences and limit of agreement ratio based on Bland–Altman analysis were used to examine agreement between estimated and actual prescribed durations.

**Results:**

rWTD models without adjustment for the quantity of dispensed medicine performed well only in the most homogenous scenario. In scenarios with greater data variability, performance improved significantly when adjusted for the quantity of dispensed medicine. Relative difference decreased from ≥ 65% in models without covariates to ≤ 20% with covariates, and the limit of agreement ratio decreased from ≥ 36.8 in models without covariates to ≤ 5.3 with covariates. Stratification analysis by subclass of the AHT medicines provided similar results.

**Conclusions:**

The study demonstrated that as data variability increased, the accuracy of the rWTD estimations decreased. However, the rWTD can produce good estimates (relative difference from 0% to 28%) of prescribed duration for AHT medicines, with the highest accuracy in the model adjusting for the quantity of dispensed medicine or stratification of the data with a relative difference less than 20% and the limit of agreement ratio less than 5.3 for the estimation at the 90th percentile of inter‐arrival density. Since this validation was limited to antihypertensive medicines, generalizing the finding to other chronic‐use medicines should be undertaken with caution.

**Summary**

- This study is among the earliest to assess the level of agreement between estimated and actual prescribed durations using real‐world data.
- The rWTD method with adjustment for quantity of dispensed medicine can be considered for estimating AHT prescribed duration in pharmacoepidemiological studies.
- Performance differs depending on the variability of the administrative data used; we provide information on how different model specifications perform under different conditions.
- Further studies are needed to validate the use of rWTD for other chronic‐use medicines.

## 1 Introduction

Health administrative data, such as medicine dispensing records, can provide invaluable insights into real‐world medicine use [1]. However, these datasets often lack crucial information on the prescribed duration of a medicine, which is necessary to estimate medicine's exposure in pharmacoepidemiological research [1, 2].

In the absence of actual prescribed duration information, several common methods such as fixed‐time windows, estimation of medicine coverage, and the prescription 2 drug use period (PRE2DUP) can be used to estimate the duration of exposure [3]. Each of these methods relies on decision rules and assumptions that may not be practical for longitudinal studies, as recommendations on doses may change over time. For example, the fixed‐time window method assumes prescribed durations last for a fixed time period, such as the maximum duration of a dispensing, without taking into account other characteristics such as the quantity dispensed [4, 5]. In the estimation of medicine coverage method, the duration of a single prescription is estimated based on dispensing history, accounting for accumulated dose and the elapsed time from previous dispensings [6]. Similarly, the PRE2DUP method estimates duration based on dispensing patterns such as regularity and periods when medicines are used or not used [7]. In contrast to these methods, the parameter waiting time distribution (WTD) is a data‐driven method estimating prescribed duration based solely on observed dispensing patterns [8, 9]. A recent extension of the WTD, called reverse WTD (rWTD) with multiple random index dates, allows for the inclusion of covariates such as quantity dispensed and other characteristics (e.g., age and sex) while also accounting for medicine stockpiling [3, 10, 11]. The rWTD method is most useful for medicines used in chronic disease settings [12].

Although the rWTD method has been recommended to estimate the prescribed duration [3, 9], validation of this method has been primarily limited to simulated data [3, 10, 11]. While these simulations for the parametric ordinary and reverse WTD have demonstrated low relative biases (−0.65%–6.64%) and high coverage (92.0%–95.3%) in the presence of seasonal stockpiling, their ability to reflect real‐world variability remains limited. Simulated data are typically generated under controlled conditions with predefined assumptions about dispensing patterns, patient adherence, and prescribing behaviors. However, in real‐world settings, factors such as variations in patient adherence, prescriber habits, and irregular refill patterns introduce additional complexity that simulations may not fully account for [11]. Thus, evaluating the performance of this method using real‐world data is essential to provide robust evidence informing its application in pharmacoepidemiology. This study aimed to validate the performance of the rWTD approach in estimating prescribed duration against actual prescribed duration recorded in the Rotterdam Study medicine data under different levels of data variability. The hypothesis was that the performance of the rWTD may diminish when complexity in data structure (i.e., the number of medicines and amount of variation in the dispensing intervals) increases. Understanding how data structure impacts precision will enable researchers to make informed decisions between pragmatism and precision—whether to input all data into the model for a useful outcome or to repeat it across smaller, homogeneous datasets for sufficient accuracy, a potentially time‐intensive task when estimating multiple medications.

The Rotterdam Study was selected because it recorded actual prescribed duration—a critical detail often missing in other datasets, making it ideal for validating prescription duration estimates [13, 14]. In this study, antihypertensives (AHTs) were used as a “case study” or exemplar of lifelong therapy. They are a good choice because they have a uniform waiting‐time distribution, which aligns well with parametric WTD models. AHTs are among the most commonly prescribed medicines, meaning our findings could serve as a valuable reference for future research on dose‐duration exposure or treatment adherence for AHTs and other medicines used chronically. In addition, this study is also a part of the large project examining the association between the long‐term use of a calcium channel blocker—a common AHT—and breast cancer risk [15].

## 2 Methods

### 2.1 Brief Review of Waiting Time Distribution Approach

Hallas et al. [16] initially proposed a waiting time graph to illustrate the distribution of intervals between the beginning of a pre‐defined observation window (e.g., index date) to the first dispensing. This graph visually conveys utilization patterns for a medicine such as prevalence and incidence rates and thus reveals the prescribing pattern. Building on this approach, Pottegård and Hallas [12] developed the “cumulative waiting time distribution graph,” which allows direct estimation of prescribed duration by identifying the number of days corresponding to a specified cumulative percentage (e.g., 80%) of prevalent users who have their first dispensing within the observation window. This approach is refined by focusing only on prevalent users—individuals with at least one dispensing of the same medication in the prior year—which reduces variation from new users and enhances estimation accuracy. The 80% cumulative percentage of the graphical distribution was recommended to use as the cut‐off for identifying the estimated prescribed duration from routinely collected medicine data [12]. This cut‐off has subsequently been adopted widely in literature on WTD methods [10, 11, 17, 18].

The graphical WTD approach is limited by its reliance on visual estimation, reducing its precision for estimating prescribed duration. To address this issue, Støvring et al. developed the parametric ordinary WTD, which applied automated estimation of prescribed duration using parametric models such as log‐normal or Weibull [10]. Unlike the graphical WTD approach, which primarily uses forward recurrence density (FRD) to measure time from the index date to the first dispensing, the parametric model transforms WTD into inter‐arrival density (IAD) function, which captures the time between consecutive dispensings within the same observation window. This transformation to IAD makes the parametric model more suitable for determining the duration of a single dispensing [10]. Similar to the graphical WTD method [12], the parametric model requires a substantial proportion of prevalent users; thus, the model is less robust for new medicines or medications with intermittent use [17]. The parametric ordinary WTD theoretically allows for the inclusion of covariates such as dispensing factors like quantity of dispensed medicine. However, the quantity of dispensed medicine of a given dispensing does not directly influence the distribution of time intervals from the index date to that dispensing. It is the quantity of dispensed medicine of the previous dispensing that impacts the estimation of the WTD [17]. The ordinary WTD method, though informative, requires a significant follow‐up period, typically at least 12 months, to accurately separate prevalent users from incident users. This necessity limits its use in estimating real‐time prevalence. Additionally, the ordinary WTD cannot estimate the cessation rate, an essential measure for interpreting shifts in medicine use patterns over time [19]. To address the limitations of the ordinary WTD, the rWTD redefines the intervals by measuring time from the last dispensing to the index date (e.g., end of the observation window) using backward recurrence density (BRD) [8, 19]. This reverse structure allows for real‐time prevalence estimation at the index date and can estimate the stopping fractions, particularly when it effectively differentiates between prevalent users and those who have discontinued treatment. Also, rWTD enables specific dispensing factors, like quantity of dispensed medicine, to directly influence the estimated interval, thus enhancing the model's precision in estimating the prescribed duration in settings where covariates are crucial.

Both parametric ordinary and rWTD models assume that patients initiate treatment at random intervals, with consistent dispensing rates over time. However, this assumption is violated when the dispensing data include issues such as medication stockpiling or seasonal fluctuations influenced by healthcare policies, seasonal health patterns, and/or variations in cultural practices. In countries like Norway and Australia, stockpiling behavior is common when patients reach a certain threshold for subsidized medicine [20, 21]. This leads to a surge in dispensing at the end of the year (e.g., November/December) as patients “stock up” on discounted medicines, followed by a sharp decline in January when out‐of‐pocket costs are reset to the start of the calendar year [20, 21]. Thus, setting 1st January as the index date under these conditions may result in shorter estimated duration with the rWTD or longer duration with the ordinary WTD. To obtain stable estimates when stockpiling or seasonal variation is present, a random index date should be selected within a pre‐defined sampling window (e.g., one calendar year) for each patient [11]. This sampling creates an observation window that extends from the random index date to an end date, ensuring that each observation window matches the sampling window in length. The total data window therefore must be at least twice the sampling window length to account for the variability introduced by randomly chosen index dates. When applying the parametric ordinary or rWTD model with a random index date, it is essential to set a random seed in statistical software such as R or Stata to ensure reproducible results. Bødkergaard et al. [18] recommend using a minimum of five random index dates within the sampling window to improve efficiency and precision, particularly when multiple covariates are included.

The parametric ordinary or rWTD analyses can be implemented using the Stata package ‘wtdttt’ [22]. We applied rWTD model with 10 random index dates to estimate the duration of AHT prescriptions in our dataset. The specifics of this approach and its application are detailed in the Section 2.5.

### 2.2 Study Setting

This study used data from the Rotterdam Study, a prospective cohort study that recruited persons 45 years and over living in the Ommoord district, Rotterdam, the Netherlands. The Rotterdam Study includes three cohorts, RS‐I, RS‐II, and RS‐III, which recruited participants in 1989, 2000, and 2006, respectively. By the end of 2008, the study consisted of 14 926 participants [13, 14]. Participants from the three Rotterdam cohorts had dispensing records from all pharmacies in the study area linked from 1991 onwards. The Rotterdam Study's dispensing data were chosen for this research due to its inclusion of real‐world prescribed duration for each dispensing record—a critical detail often missing in other dispensing datasets.

### 2.3 Data Selection and Preparation

The current study included 3327 participants of the Rotterdam Study who had AHT dispensings between January 1, 2018 and December 31, 2019. The study's medicine dispensing data included, for each prescription: a de‐identified unique person identifier, the quantity of dispensed medicine, the actual prescribed durations, the fifth level Anatomical Therapeutic Chemical (ATC) code, and the total defined daily dose (DDD). The ATC classification system provides classifications of medicines at various levels of detail [23]. The ATC 5 codes specify the exact medicines used, such as losartan with the ATC 5 code of “C09CA01,” while the ATC 2 codes refer to broader subclasses of medicines, such as “agents acting on the renin‐angiotensin system” with the ATC 2 code of “C09.” AHT medicines were classified into five subclasses: beta‐blocking agents (BB) (ATC code C07), (ii) calcium channel blockers (CCBs) (C08); (iii) diuretics (C03), (iv) agents acting on the renin‐angiotensin system (C09), or (v) other AHTs not included in these classes (C02). Since this study focused on validating prescribed durations, prescriptions with missing data or extreme values (e.g., values above the 99th percentile) in actual prescribed durations and quantities of dispensed medicines were excluded.

### 2.4 Scenarios With Varying Levels of Data Variability

**FIGURE 1:**
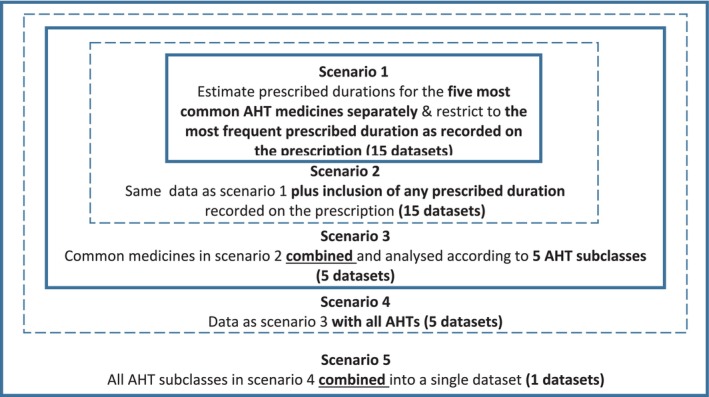
Scenario setting. Solid box: Data with the same specification. Dash box: Data with the same specification but in either separate or combined subsets. AHT: Antihypertensive.

The person‐level medicine data were structured into five scenarios representing different variability contexts, from a simple context with a single medicine and regular dispensing (Scenario 1) to scenarios with greater amounts of variability in terms of multiple medicines and various prescribed durations to test the accuracy of rWTD models in estimating the prescribed duration of a medicine (Figure 1). These scenarios also correspond to different levels of information potentially available in administrative medicine data. Therefore, the findings provide valuable information for potential users of rWTD, enabling them to make informed decisions regarding the trade‐off between analytic time and effort versus the level of accuracy in the estimates of prescription duration required for their study.

- **Scenario 1:** Evaluated the performance of the rWTD method in an idealized setting where dispensing periods were highly homogeneous. This scenario was designed to minimize variability in dispensing parameters by including only records of the three most commonly prescribed medicines within each AHT subclass. The scenario included 15 medicines; separate datasets and analyses were used. For each of these medicines, the data was further restricted to only include records with the most frequent prescribed duration as detailed on the prescription. The rWTD models were applied at the ATC 5 level to estimate the prescribed duration for each medicine.
- **Scenario 2:** Removed the restriction on actual prescribed durations included as detailed in Scenario 1 but remained focused on the same 15 medicines. As in Scenario 1, the rWTD models were run separately for each medicine at the ATC 5 level to estimate the prescribed durations. This approach allowed for testing the models' accuracy in more realistic, less controlled conditions, which may better reflect real‐world scenarios.
- **Scenario 3:** Used the same data in Scenario 2 but medicines in the same AHT subclass were grouped together into one data file, resulting in five datasets. This scenario introduced additional variation across the common AHT medicines within subclass. The rWTD models were then applied separately to estimate the prescribed duration for each subclass at the ATC 2 level (e.g., C07 for BB, C08 for CCB, C03 for diuretics, C09 for renin‐angiotensin system agents and C02 for other AHTs). This approach is particularly useful for larger pharmacoepidemiological datasets, where running separate rWTD models for each AHT medicine could be time‐consuming due to the large number of models required.
- **Scenario 4:** Extended Scenario 3 to include all medicines within each AHT subclass, making use of complete medicine dispensing data. In this scenario, only information on AHT subclass or ATC 2 codes were required, rather than the specific medicine names or ATC 5 codes. Similar to Scenario 3, the WTD models were run to estimate durations for each of the five AHT subclasses at ATC 2 level. This scenario assessed whether the models performed effectively when working with medicine dispensing datasets that contain only the ATC 2 codes, without more specific medicine‐level data.
- **Scenario 5:** Used the complete medicine dispensing data as in Scenario 4 with all AHT subclasses combined into a single data file consisting of all AHT medicines. The rWTD model was applied to the entire dataset to estimate the prescribed durations. This scenario was designed to determine whether the model's accuracy began to diminish when applied to a whole large dataset.

### 2.5 Analysis

Firstly, descriptive statistics were performed on participant sociodemographic characteristics and number of prescriptions across the five main AHT subclasses.

The rWTD with a log‐normal prevalent component (backward recurrence density) and parameters μ and *σ* to estimate a single Log‐Normal IAD with 10 random index dates was used [18]. Separate models with and without covariates of the quantity of dispensed medicine and ATC level 5 codes in a single prescription were conducted for each scenario where appropriate. The IAD was estimated at the 50th, 60th, 70th, 80th, and 90th percentiles. The data window was from January 1, 2018 to December 31, 2019 (2δ), and the sampling window for 10 random index dates was from January 1, 2019 to December 31, 2019 (δ). The package “wtdttt” was installed in Stata to run the rWTD models [22].

Bland–Altman analysis was used to assess the agreement between the estimated prescribed durations based on rWTD and the actual prescribed durations of single prescriptions [24]. Similar to the comparison method performed by Thrane et al. [25], for each patient, the estimated prescribed duration was subtracted from the actual prescribed duration on a logarithmic scale, representing the ‘average difference δ’ between the estimated and actual prescribed durations. The relative difference in percentage was then calculated as (exp(δ) − 1). The “limits of agreement” was also computed on the logarithmic scale as δ ± 1.96SD. To assess the performance of rWTD, the variation between estimated prescribed durations and actual prescribed durations was presented by the ratio of the upper to the lower limit of agreement, with both limits transformed back to the original scale. A lower limits of agreement ratio or a lower relative difference suggests a more precise estimation of the duration for a single prescription. In a sensitivity analysis, we used the rWTD model to estimate prescription durations for a single medicine, C07AB02 (metoprolol) as example, across different sample sizes including 11 733, 5787, 2857, 894, and 445 prescriptions. The samples were randomly selected from prescriptions of the medicine. All statistical analyses were conducted in Stata version 18.0 [26].

## 3 Results

The participants across all five scenarios had similar sociodemographic characteristics, with a median (±SD) age of 76 (9.1) years and 58% female. A total of 100 725 prescriptions were included in this study; RAS (*n* = 31 463, 31.2%) and diuretics (*n* = 23 824, 23.7%) were the most common AHT subclasses (Table 1).

**TABLE 1:** Characteristics of included participants and prescriptions.

	Scenario 1	Scenario 2/Scenario 3	Scenario 4/Scenario 5
Characteristics of participants			
No. of participants	1567	2966	3327
Age, median (IQR), years	76.2 (69.6–83.2)	76.2 (69.2–83.2)	75.7 (69.0–82.9)
Female, *n* (%)	919 (58.7)	1706 (57.5)	1941 (58.3)
Characteristics of prescriptions			
No. of prescriptions	16 628	77 278	100 725
AHT subclass, *n* (%)			
BB	3901 (23.5)	25 104 (32.5)	29 542 (29.3)
CCB	2044 (12.3)	12 468 (16.1)	14 941 (14.8)
RAS	3276 (19.7)	20 514 (26.6)	31 463 (31.2)
Diuretics	7171 (43.1)	18 246 (23.6)	23 824 (23.7)
Other AHT	236 (1.4)	946 (1.2)	955 (1.0)
No. of medicines (ATC 5)	15	15	74
Frequency of dispensing per year, mean (SD)	16 (12)	17 (11)	17 (11)
Actual prescribed duration, mean (SD), days	51 (38)	43 (40)	43 (40)

Abbreviations: AHT, antihypertensive; BB, beta‐blocking agents; CCB, calcium‐channel blockers; RAS, agents acting on the renin‐angiotensin system.

Results from the univariate rWTD models are presented in Table 2. The relative differences between the estimated and actual prescribed duration increased as the variability of the data and dispensing variation increased across the five scenarios. The relative differences across IAD percentiles ranged between 56%–75% for Scenario 2–5, while Scenario 1 ranged within 26%, with the smallest relative difference of 1% recorded at the 70th percentile. Similar patterns were observed for the limit of agreement and limit of agreement ratio.

**TABLE 2:** Comparison of estimated prescribed durations based on reverse waiting time distribution with multiple random index dates in univariate models versus actual prescribed durations.

rWTD estimation	Actual prescribed duration (days) median (IQR)	Estimated prescribed duration (days) median (IQR)	Relative difference (%)	Limit of agreements (%)	Limit of agreement ratio
Scenario 1: Estimate prescribed durations for each AHT medicine in the data of common AHT medicines and common actual prescribed durations					
50th percentile of IAD	30 (12–90)	10.9 (8.2–89.8)	26%	−40 to 161	4.265
60th percentile of IAD	30 (12–90)	14.3 (9.0–95.3)	13%	−43 to 127	3.943
70th percentile of IAD	30 (12–90)	19.2 (10.1–101.1)	1%	−50 to 103	4.101
80th percentile of IAD	30 (12–90)	27.2 (11.4–108.6)	−12%	−60 to 95	4.797
90th percentile of IAD	30 (12–90)	43.8 (13.6–119.9)	−26%	−73 to 97	7.099
Scenario 2: Estimate prescribed durations for each AHT medicine in the data of common AHT medicines and varying actual prescribed durations					
50th percentile of IAD	19 (12–90)	77.2 (71.0–80.5)	−56%	−94 to 206	48.463
60th percentile of IAD	19 (12–90)	82.5 (78.1–85.6)	−60%	−94 to 164	43.086
70th percentile of IAD	19 (12–90)	88.7 (86.6–90.9)	−64%	−94 to 153	39.837
80th percentile of IAD	19 (12–90)	96.8 (96.3–97.5)	−68%	−95 to 95	38.306
90th percentile of IAD	19 (12–90)	107.4 (106.0–109.2)	−73%	−96 to 63	36.833
Scenario 3: Estimate prescribed durations for each AHT subclass in the data of common AHT medicines and varying actual prescribed durations					
50th percentile of IAD	19 (12–90)	75.5 (75.5–79.8)	−61%	−94 to 146	40.467
60th percentile of IAD	19 (12–90)	81.1 (81.1–85.1)	−65%	−94 to 123	39.837
70th percentile of IAD	19 (12–90)	87.5 (87.5–91.2)	−68%	−95 to 102	39.526
80th percentile of IAD	19 (12–90)	95.6 (95.6–98.8)	−71%	−95 to 80	39.526
90th percentile of IAD	19 (12–90)	110.4 (108.0–110.4)	−65%	−96 to 55	40.151
Scenario 4: Estimate prescribed durations for each AHT subclass in the data of all AHT medicines and varying actual prescribed durations					
50th percentile of IAD	19 (12–90)	79.9 (76.9–87.7)	−62%	−94 to 142	41.106
60th percentile of IAD	19 (12–90)	84.7 (82.6–86.2)	−65%	−95 to 122	40.467
70th percentile of IAD	19 (12–90)	90.2 (89.1–91.3)	−68%	−95 to 103	40.308
80th percentile of IAD	19 (12–90)	97.0 (97.0–97.7)	−71%	−95 to 83	40.467
90th percentile of IAD	19 (12–90)	107.3 (107.3–110.3)	−75%	−96 to 61	41.268
Scenario 5: Estimate prescribed durations for all AHT (single estimates) in the data of all AHT medicines and varying actual prescribed durations					
50th percentile of IAD	19 (12–90)	72.5 (72.5–72.5)	−62%	−94 to 143	40.626
60th percentile of IAD	19 (12–90)	78.2 (78.2–78.2)	−65%	−94 to 125	40.626
70th percentile of IAD	19 (12–90)	84.7 (84.7–84.7)	−67%	−95 to 108	40.467
80th percentile of IAD	19 (12–90)	93.0 (93.0–93.0)	−70%	−95 to 89	40.546
90th percentile of IAD	19 (12–90)	106.0 (106.0–106.0)	−74%	−96 to 66	40.626

Abbreviations: AHT, antihypertensive; ATC, anatomical therapeutic chemical; IAD, inter‐arrival density; rWTD, reverse waiting time distribution.

Table 3 shows the results from rWTD models adjusted for the quantity of dispensed medicine. The relative differences and the limit of agreement ratios were significantly reduced compared with those observed in the univariate models, particularly in Scenarios 2–5. The smallest relative differences were observed at the estimates based on the 60th percentile of IAD (6%) in Scenario 1, and the 90th percentile of IAD (ranging from 0% to 7%) in Scenarios 2–5. The limit of agreement ratio was relatively consistent across all percentiles of IAD in all scenarios (ranging from 2.6 to 6.1). In Table 4, the rWTD models with further adjustments for ATC 5 level codes in Scenarios 3, 4, and 5 yielded similar patterns in relative differences and limit of agreement ratios as those observed in Table 4. Estimates based on the 90th percentile of IAD produced the smallest relative difference (ranging from 3% to 8%) and a relatively low limit of agreement ratio of 3.5.

**TABLE 3:** Comparison of estimated prescribed durations based on reverse waiting time distribution with multiple random index dates in models adjusting for quantity of dispensed medicine versus actual prescribed durations.

rWTD estimation	Actual prescribed duration (days) median (IQR)	Estimated prescribed duration (days) median (IQR)	Relative difference (%)	Limit of agreements (%)	Limit of agreement ratio
Scenario 1: Estimate prescribed durations for each AHT medicine in the data of common AHT medicines and common actual prescribed durations					
50th percentile of IAD	30 (12–90)	31.6 (8.8–92.5)	9%	−64 to 278	2.642
60th percentile of IAD	30 (12–90)	32.5 (9.0–95.5)	6%	−65 to 274	2.727
70th percentile of IAD	30 (12–90)	35.7 (9.6–101.6)	−16%	−67 to 249	2.654
80th percentile of IAD	30 (12–90)	39.8 (10.2–109.0)	−11%	−70 to 225	2.685
90th percentile of IAD	30 (12–90)	46.3 (11.1–123.2)	−20%	−74 to 197	2.696
Scenario 2: Estimate prescribed durations for each AHT medicine in the data of common AHT medicines and varying actual prescribed durations					
50th percentile of IAD	19 (12–90)	14.5 (9.3–90.2)	13%	−54 to 175	5.998
60th percentile of IAD	19 (12–90)	14.8 (8.0–92.3)	16%	−46 to 148	4.540
70th percentile of IAD	19 (12–90)	15.4 (9.0–95.2)	5%	−56 to 161	6.021
80th percentile of IAD	19 (12–90)	16.4 (9.6–98.9)	0%	−59 to 145	6.116
90th percentile of IAD	19 (12–90)	17.0 (9.0–103.7)	−1%	−54 to 114	4.667
Scenario 3: Estimate prescribed durations for each AHT subclass in the data of common AHT medicines and varying actual prescribed durations					
50th percentile of IAD	19 (12–90)	14.3 (8.0–90.5)	28%	−33 to 144	3.660
60th percentile of IAD	19 (12–90)	14.6 (8.1–92.7)	24%	−35 to 138	3.660
70th percentile of IAD	19 (12–90)	15.1 (8.3–95.9)	19%	−38 to 130	3.689
80th percentile of IAD	19 (12–90)	15.7 (8.3–99.7)	6%	−52 to 137	4.949
90th percentile of IAD	19 (12–90)	16.6 (8.9–105.3)	0%	−57 to 129	5.270
Scenario 4: Estimate prescribed durations for each AHT subclass in the data of all AHT medicines and varying actual prescribed durations					
50th percentile of IAD	19 (12–90)	14.1 (7.5–90.3)	26%	−35 to 145	3.776
60th percentile of IAD	19 (12–90)	14.4 (7.6–92.4)	23%	−37 to 140	3.791
70th percentile of IAD	19 (12–90)	15.1 (7.9–95.4)	19%	−39 to 132	3.806
80th percentile of IAD	19 (12–90)	15.9 (8.3–99.1)	14%	−42 to 123	3.836
90th percentile of IAD	19 (12–90)	16.9 (8.6–104.4)	7%	−46 to 111	3.897
Scenario 5: Estimate prescribed durations for all AHT (single estimates) in the data of all AHT medicines and varying actual prescribed durations					
50th percentile of IAD	19 (12–90)	14.0 (7.5–87.0)	28%	−32 to 141	3.547
60th percentile of IAD	19 (12–90)	14.4 (7.6–89.6)	23%	−34 to 129	3.451
70th percentile of IAD	19 (12–90)	15.0 (8.3–93.2)	19%	−37 to 124	3.547
80th percentile of IAD	19 (12–90)	16.0 (8.6–98.9)	13%	−41 to 114	3.603
90th percentile of IAD	19 (12–90)	17.0 (9.0–106.4)	4%	−45 to 95	3.533

Abbreviations: AHT, antihypertensive; ATC, anatomical therapeutic chemical; IAD, inter‐arrival density; rWTD, reverse waiting time distribution.

**TABLE 4:** Comparison of estimated prescribed durations based on reverse waiting time distribution with multiple random index dates in models adjusting for quantity of dispensed medicine and ATC level 5 codes versus actual prescribed durations.

rWTD estimation	Actual prescribed duration (days) median (IQR)	Estimated prescribed duration (days) median (IQR)	Relative difference (%)	Limit of agreements (%)	Limit of agreement ratio
Scenario 3: Estimate prescribed durations for each AHT subclass in the data of common AHT medicines and varying actual prescribed durations					
50th percentile of IAD	19 (12–90)	14.2 (7.4–89.6)	27%	−32 to 137	3.492
60th percentile of IAD	19 (12–90)	14.5 (7.5–91.9)	24%	−33 to 130	3.437
70th percentile of IAD	19 (12–90)	15.0 (7.8–94.6)	20%	−36 to 124	3.519
80th percentile of IAD	19 (12–90)	15.6 (8.2–98.8)	15%	−39 to 115	3.519
90th percentile of IAD	19 (12–90)	16.5 (8.7–102.7)	8%	−42 to 104	3.547
Scenario 4: Estimate prescribed durations for each AHT subclass in the data of all AHT medicines and varying actual prescribed durations					
50th percentile of IAD	19 (12–90)	14.2 (7.4–89.7)	23%	−33 to 126	3.357
60th percentile of IAD	19 (12–90)	14.6 (8.1–91.7)	21%	−34 to 122	3.384
70th percentile of IAD	19 (12–90)	15.4 (8.8–95.9)	16%	−37 to 113	3.384
80th percentile of IAD	19 (12–90)	15.9 (9.1–99.6)	11%	−40 to 105	3.410
90th percentile of IAD	19 (12–90)	17.7 (10.6–105.3)	3%	−59 to 167	3.437
Scenario 5: Estimate prescribed durations for all AHT (single estimates) in the data of all AHT medicines and varying actual prescribed durations					
50th percentile of IAD	19 (12–90)	13.9 (7.7–87.0)	29%	−28 to 132	3.228
60th percentile of IAD	19 (12–90)	14.2 (7.8–89.1)	26%	−30 to 129	3.279
70th percentile of IAD	19 (12–90)	14.8 (8.2–92.0)	21%	−33 to 120	3.305
80th percentile of IAD	19 (12–90)	15.8 (8.8–97.8)	13%	−38 to 106	3.318
90th percentile of IAD	19 (12–90)	17.0 (9.4–104.4)	4%	−45 to 97	3.589

Abbreviations: AHT, antihypertensive; ATC, anatomical therapeutic chemical; IAD, inter‐arrival density; rWTD, reverse waiting time distribution.

**FIGURE 2:**
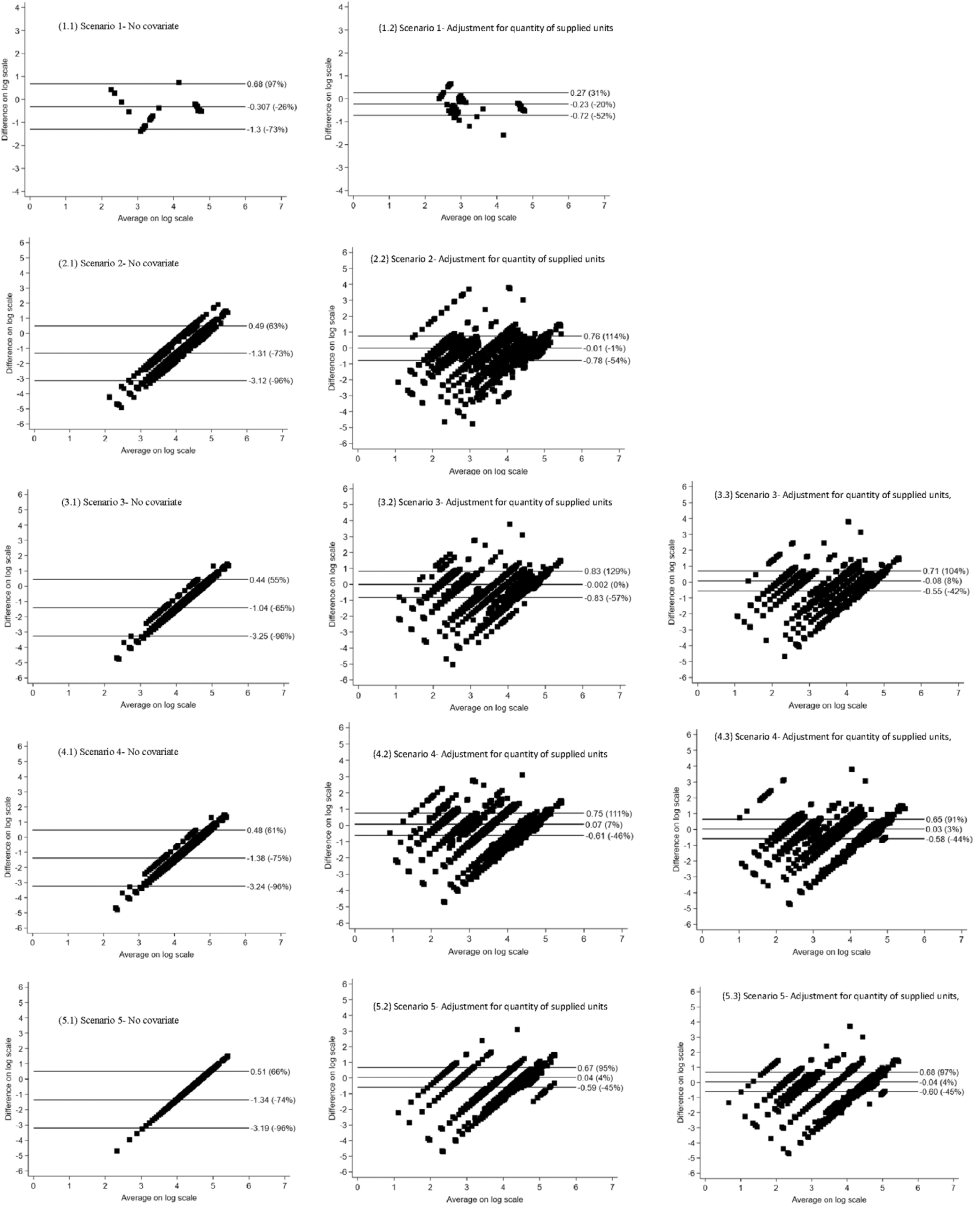
Bland–Altman plots comparing estimated prescribed durations based on the 90th percentile of inter‐arrival density with actual prescribed durations. AHT, antihypertensive; ATC, anatomical therapeutic chemical. Scenario 1: Estimate prescribed durations for each AHT medicine in the data of common AHT medicines and common actual prescribed durations. Scenario 2: Estimate prescribed durations for each AHT medicine in the data of common AHT medicines and varying actual prescribed durations. Scenario 3: Estimate prescribed durations for each AHT subclass in the data of common AHT medicines and varying actual prescribed durations. Scenario 4: Estimate prescribed durations for each AHT subclass in the data of all AHT medicines and varying actual prescribed durations. Scenario 5: Estimate prescribed durations for all AHT (single estimates) in the data of all AHT medicines and varying actual prescribed durations.

**FIGURE A1:**
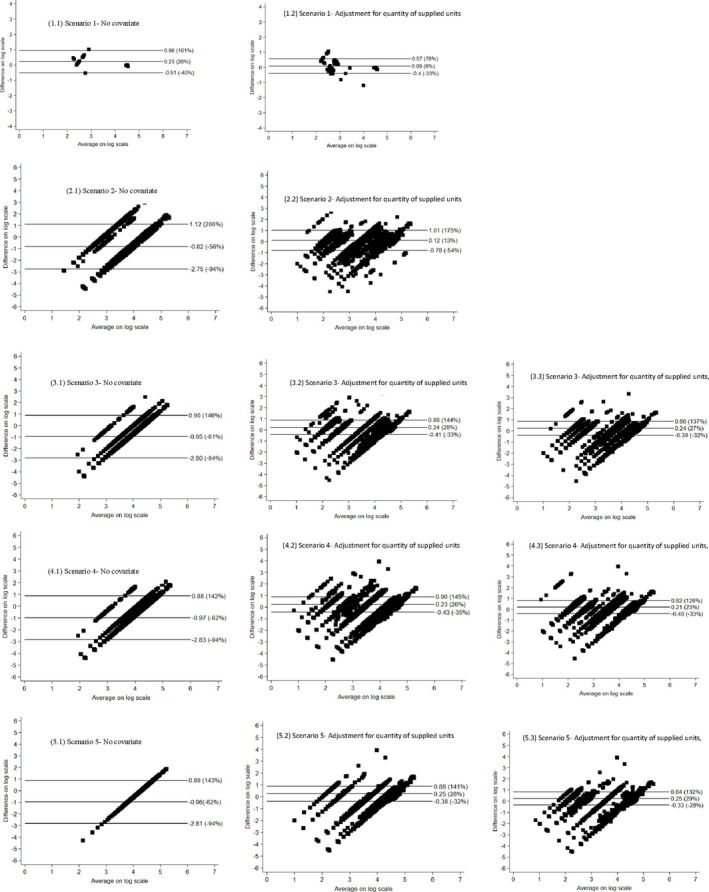
Bland–Altman plots comparing estimated prescribed durations based on the 50th percentile of inter‐arrival density with actual prescribed durations. AHT, antihypertensive; ATC, anatomical therapeutic chemical. Scenario 1: Estimate prescribed durations for each AHT medicine in the data of common AHT medicines and common actual prescribed durations. Scenario 2: Estimate prescribed durations for each AHT medicine in the data of common AHT medicines and varying actual prescribed durations. Scenario 3: Estimate prescribed durations for each AHT subclass in the data of common AHT medicines and varying actual prescribed durations. Scenario 4: Estimate prescribed durations for each AHT subclass in the data of all AHT medicines and varying actual prescribed durations. Scenario 5: Estimate prescribed durations for all AHT (single estimates) in the data of all AHT medicines and varying actual prescribed durations.

**FIGURE A2:**
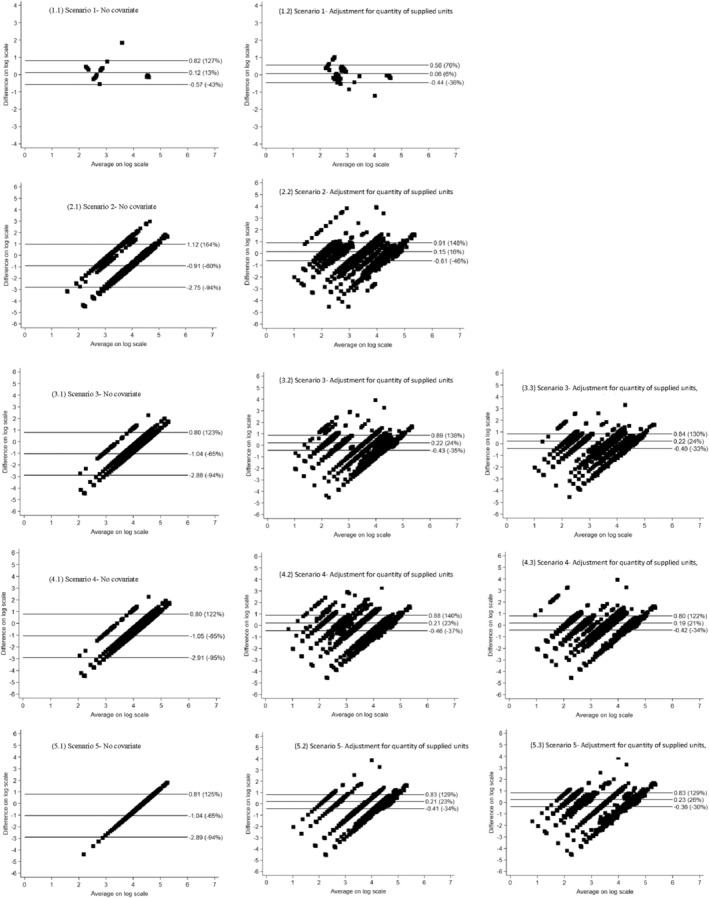
Bland–Altman plots comparing estimated prescribed durations based on the 60th percentile of inter‐arrival density with actual prescribed durations. AHT, antihypertensive; ATC, anatomical therapeutic chemical. Scenario 1: Estimate prescribed durations for each AHT medicine in the data of common AHT medicines and common actual prescribed durations. Scenario 2: Estimate prescribed durations for each AHT medicine in the data of common AHT medicines and varying actual prescribed durations. Scenario 3: Estimate prescribed durations for each AHT subclass in the data of common AHT medicines and varying actual prescribed durations. Scenario 4: Estimate prescribed durations for each AHT subclass in the data of all AHT medicines and varying actual prescribed durations. Scenario 5: Estimate prescribed durations for all AHT (single estimates) in the data of all AHT medicines and varying actual prescribed durations.

**FIGURE A3:**
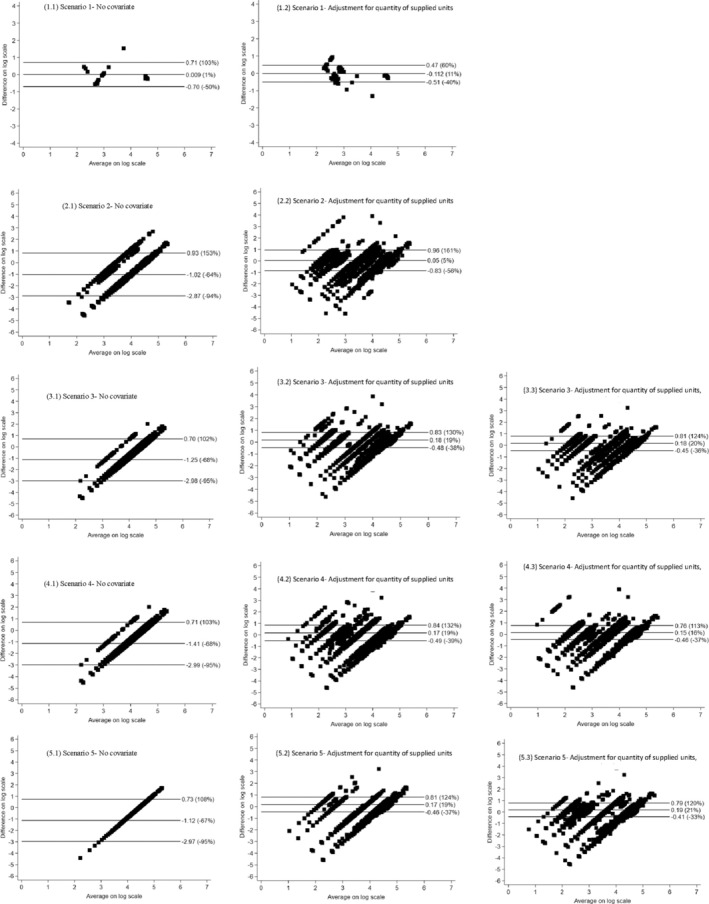
Bland–Altman plots comparing estimated prescribed durations based on the 70th percentile of inter‐arrival density with actual prescribed durations. AHT, antihypertensive; ATC, anatomical therapeutic chemical. Scenario 1: Estimate prescribed durations for each AHT medicine in the data of common AHT medicines and common actual prescribed durations. Scenario 2: Estimate prescribed durations for each AHT medicine in the data of common AHT medicines and varying actual prescribed durations. Scenario 3: Estimate prescribed durations for each AHT subclass in the data of common AHT medicines and varying actual prescribed durations. Scenario 4: Estimate prescribed durations for each AHT subclass in the data of all AHT medicines and varying actual prescribed durations. Scenario 5: Estimate prescribed durations for all AHT (single estimates) in the data of all AHT medicines and varying actual prescribed durations.

**FIGURE A4:**
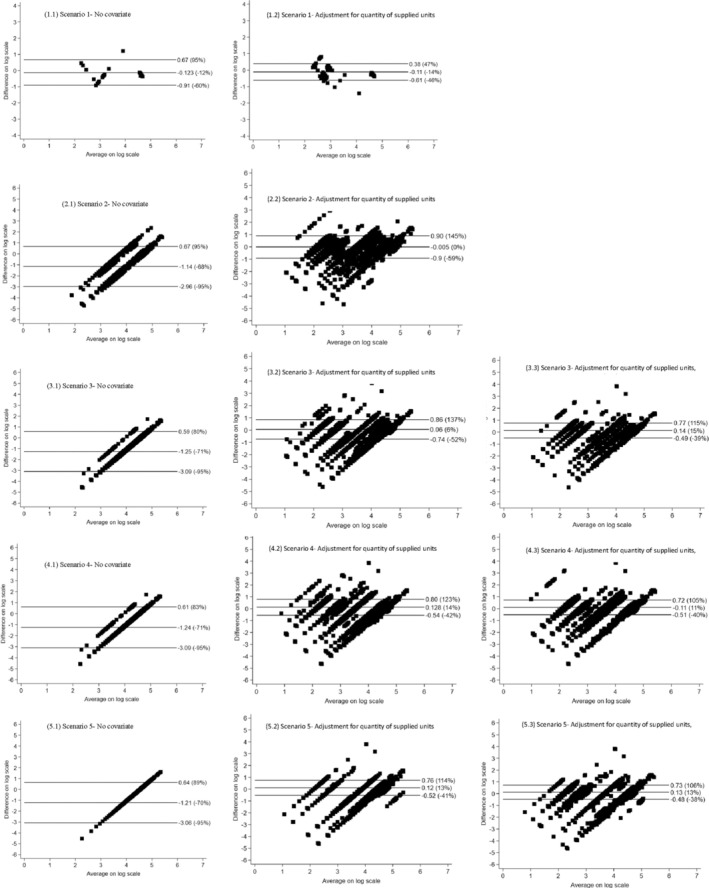
Bland–Altman plots comparing estimated prescribed durations based on the 80th percentile of inter‐arrival density with actual prescribed durations. AHT, antihypertensive; ATC, anatomical therapeutic chemical. Scenario 1: Estimate prescribed durations for each AHT medicine in the data of common AHT medicines and common actual prescribed durations. Scenario 2: Estimate prescribed durations for each AHT medicine in the data of common AHT medicines and varying actual prescribed durations. Scenario 3: Estimate prescribed durations for each AHT subclass in the data of common AHT medicines and varying actual prescribed durations. Scenario 4: Estimate prescribed durations for each AHT subclass in the data of all AHT medicines and varying actual prescribed durations. Scenario 5: Estimate prescribed durations for all AHT (single estimates) in the data of all AHT medicines and varying actual prescribed durations.

Given that estimates based on the 90th percentile of IAD demonstrated the smallest relative differences and limits of agreement in most scenarios, we provided Bland–Altman plots of these estimates for each of the five scenarios in Figure 2. Plots from other percentiles of IAD were available in Figures A1, A2, A3, A4. These plots graphically presented the relative difference and limit of agreement detailed in Tables 3 and 4. In Figure 2, regarding the estimates based on univariate rWTD models, Scenario 1 showed the smallest relative difference of 26%, with all data points falling within the limits of agreement and no clear pattern observed. In contrast, the plots for Scenarios 2, 3, and 4 displayed a significantly higher relative differences (≥ 65%), with parallel lines suggesting that varying quantities of medicine were dispensed. In Scenario 5, the plot indicated a relative difference of −74%, forming a straight line due to the fixed estimation from the rWTD model applied uniformly to all AHT prescriptions. When adjusting for the quantity of dispensed medicine, the data points in the plots displayed greater horizontal dispersion across all scenarios, with Scenario 3 showing the smallest relative difference of 0%. The clustering patterns in the plots of Scenarios 3, 4, and 5 improved slightly when ATC level 5 codes were further adjusted in the rWTD models.

In the sensitivity analysis, our findings revealed that accuracy declined as sample sizes decreased in models with and without adjustment for quantity of dispensed medicine, though the decline was more pronounced in the adjusted models. In these adjusted models, the smallest relative differences, 17% and −27%, with limit‐of‐agreement ratios below 3, were observed in datasets with sample sizes of 11 733 and 5787 prescriptions, respectively. Accuracy began to drop at a sample size of 2857 prescriptions (minimum relative difference = −65%, limit‐of‐agreement ratio = 7) and fell sharply when the sample size decreased below 1000 prescriptions, with limit‐of‐agreement ratios rising to at least 30 and the minimum relative difference reaching −75%. Further details were provided in Tables A1 and A2.

## 4 Discussion

This study evaluated the performance of the rWTD using multiple random index dates across different levels of data variability against the actual prescribed duration recorded in real‐world medicine data from the Rotterdam Study. With the actual prescribed duration recorded in the Rotterdam Study data, it is clear that prescriptions for the same medicine can vary in duration for the same patient over time, even for regular, long‐term treatments like AHT medicines. These differences often arise due to unrecorded reasons, such as adjustments by the prescriber based on changes in the patient's health status or logistical considerations. Therefore, it is almost unrealistic to simulate data that can mimic the variability of real‐world prescribing practices to be used for evaluating the performance of the rWTD method. This study took advantage of the Rotterdam Study data, which recorded the actual prescribed duration for each dispensing to provide for the first time an evaluation of the performance of the rWTD in estimating prescribed duration against the actual prescribed duration. This study found that the estimation accuracy level decreased as data variability increased. However, estimations were improved by adjusting for the quantity of dispensed medicine. For researchers considering using administrative data to estimate medicine use, our work highlights some key considerations. Firstly, these results demonstrated that the rWTD can be used to produce reasonable estimates of prescribed duration—critical information (but often not recorded in administrative data) [1, 2]. Secondly, these results demonstrate which specific aspects of data preparation and rWTD models are necessary under different contexts. For studies requiring high accuracy, especially in individual‐level adherence assessments, data should be refined to a detailed level—such as by medicine‐specific or ATC 5 codes—and should include the quantity of dispensed medicine. Although this approach enhances precision, it may also be highly resource‐intensive due to the extensive number of models required. In contrast, for studies focused on broader, population‐level estimates of medicine use, models could be structured by grouping medicines at the subclass level (e.g., BB, CCB). This approach can reduce model variability and execution time, offering an efficient solution for studies prioritizing general insights over granular detail.

Similar to our results, Støvring et al. [8] reported that the quantity of dispensed medicine was the strongest predictor of prescribed duration in the original rWTD model with a fixed index date for warfarin users in Denmark. Most of the previous empirical studies of WTD and rWTD used estimates based on the 80th percentile of IAD when estimating prescribed durations of non‐steroidal anti‐inflammatory drugs, warfarin, bendroflumethiazide, and levothyroxine [8, 10, 11, 18]. Our study observed that the 90th percentile of IAD performed better than the lower percentiles and thus yielded lower bias estimates for AHT prescribed duration. The choice of IAD percentile can be considered as setting a threshold for the misclassification between continued and discontinued medication use. When the 90th percentile of IAD is used to estimate the duration of a single prescription, there is a 10% likelihood of misclassifying continued users as having stopped. Since AHT medications are generally used chronically or continuously, utilizing a higher IAD percentile based on rWTD may reduce the likelihood of misclassification.

The main strength of this study is the use of actual prescribed durations from prospectively collected data in a large cohort to validate the performance of rWTD‐based approaches in administrative dispensing data. The study provides a valuable framework for determining the duration of AHT prescription in future pharmacoepidemiological studies focusing on AHTs, noting our results may not be generalizable to other medicines or across other health jurisdictions. Similar to other methods used to estimate prescribed duration, the rWTD approach does not account for variations in medicine adherence or other potential confounders, such as indication, contraindication, and disease severity, which are often missing from medicine dispensing data [3, 6, 27, 28]. Further research is required to validate the application of rWTD for other chronic‐use medications across diverse countries with varying medicine dispensation policies.

## 5 Conclusion

The study demonstrated that as data variability increased, the accuracy of the rWTD estimations decreased. However, the rWTD can produce good estimates (relative difference from 0% to 28%) of prescribed duration for AHT medicines, with the highest accuracy in the model adjusting for covariates or stratification of the data with a relative difference of less than 20% and the limit of agreement ratio of less than 5.3 for the estimation at the 90th percentile of IAD. Since AHT medications are generally used chronically or continuously, utilizing a higher IAD percentile based on rWTD may reduce the likelihood of exposure misclassification. The findings should be interpreted with caution as the study focused exclusively on AHT medicines and did not account for other unmeasured factors such as medicine adherence, indications, contraindications, or disease severity.

## Disclosure

This research used data from the Rotterdam Study. Information on the process for accessing the data can be found in the website https://www.erasmusmc.nl/en/research/core‐facilities/ergo‐the‐rotterdam‐study.

## Ethics Statement

Ethical approval was obtained from the following Human Research Ethics Committees: Curtin University (HRECs) (ref No. HRE2022‐0335). The Rotterdam Study has been approved by the Medical Ethics Committee of the Erasmus MC (registration number MEC 02.1015) and by the Dutch Ministry of Health, Welfare and Sport (Population Screening Act WBO, license number 1071272‐159521‐PG). The Rotterdam Study Personal Registration Data collection is filed with the Erasmus MC Data Protection Officer under registration number EMC1712001. The Rotterdam Study has been entered into the Dutch Trial Register (NTR; https://onderzoekmetmensen.nl) and into the WHO International Clinical Trials Registry Platform (ICTRP https://www.who.int/clinical‐trials‐registry‐platform, search portal https://trialsearch.who.int/) under shared catalogue number NL6645/NTR6831. The Rotterdam Study project persistent identifier is https://ror.org/02ac58f22.

## Consent

All participants provided written informed consent to participate in the study and to have their information obtained from treating physicians.

## Conflicts of Interest

The authors declare no conflicts of interest.

## Appendix A

**TABLE A1:** Comparison of estimated prescribed durations based on reverse waiting time distribution with multiple random index dates in univariate models versus actual prescribed durations for metoprolol (C07AB02) at different sample sizes.

rWTD estimation	Actual prescribed duration (days) median (IQR)	Estimated prescribed duration (days) median	Relative difference (%)	Limit of agreements (%)	Limit of agreement ratio
C07AB02 (*n* = 11 733)					
50th percentile of IAD	19 (12–90)	82	−65%	−95 to 118	39.837
60th percentile of IAD	19 (12–90)	87	−67%	−95 to 108	39.837
70th percentile of IAD	19 (12–90)	92	−69%	−95 to 93	39.837
80th percentile of IAD	19 (12–90)	99	−71%	−95 to 80	39.837
90th percentile of IAD	19 (12–90)	109	−74%	−96 to 63	39.837
C07AB02 (*n* = 5787)					
50th percentile of IAD	19 (12–90)	74	−62%	−94 to 136	39.837
60th percentile of IAD	19 (12–90)	86	−68%	−95 to 101	39.837
70th percentile of IAD	19 (12–90)	102	−73%	−96 to 70	39.837
80th percentile of IAD	19 (12–90)	124	−78%	−96 to 40	39.837
90th percentile of IAD	19 (12–90)	163	−83%	−97 to 6	39.837
C07AB02 (*n* = 2857)					
50th percentile of IAD	19 (12–90)	91	−70%	−95 to 90	38.306
60th percentile of IAD	19 (12–90)	103	−73%	−96 to 65	38.306
70th percentile of IAD	19 (12–90)	119	−78%	−95 to 43	38.306
80th percentile of IAD	19 (12–90)	141	−80%	−97 to 22	38.306
90th percentile of IAD	19 (12–90)	177	−85%	−98 to −3	38.306
C07AB02 (*n* = 894)					
50th percentile of IAD	18 (12–90)	91	−70%	−95 to 80	38.759
60th percentile of IAD	18 (12–90)	103	−75%	−96 to 60	38.759
70th percentile of IAD	18 (12–90)	117	−78%	−96 to 40	38.759
80th percentile of IAD	18 (12–90)	137	−80%	−97 to 21	38.759
90th percentile of IAD	18 (12–90)	169	−83%	−97 to −2	38.759
C07AB02 (*n* = 445)					
50th percentile of IAD	18 (12–90)	177	−85%	−98 to −4	40.626
60th percentile of IAD	18 (12–90)	103	−74%	−96 to 65	40.626
70th percentile of IAD	18 (12–90)	199	−87%	−98 to −15	40.626
80th percentile of IAD	18 (12–90)	215	−88%	−98 to −21	40.626
90th percentile of IAD	18 (12–90)	238	−89%	−98 to −29	40.626

Abbreviations: IAD, inter‐arrival density; rWTD, reverse waiting time distribution.

**TABLE A2:** Comparison of estimated prescribed durations based on reverse waiting time distribution with multiple random index dates in models adjusting for quantity of dispensed medicine versus actual prescribed durations for metoprolol (C07AB02) at different sample sizes.

rWTD estimation	Actual prescribed duration (days) median (IQR)	Estimated prescribed duration (days) median (IQR)	Relative difference (%)	Limit of agreements (%)	Limit of agreement ratio
C07AB02 (*n* = 11 733)					
50th percentile of IAD	19 (12–90)	14 (7–90)	31%	−24 to 127	2.997
60th percentile of IAD	19 (12–90)	15 (7–92)	28%	−25 to 123	2.997
70th percentile of IAD	19 (12–90)	15 (8–94)	26%	−27 to 116	2.997
80th percentile of IAD	19 (12–90)	16 (8–97)	22%	−30 to 110	2.997
90th percentile of IAD	19 (12–90)	16 (8–101)	17%	−32 to 101	2.997
C07AB02 (*n* = 5787)					
50th percentile of IAD	19 (12–90)	30 (15–138)	−27%	−54 to 16	2.464
60th percentile of IAD	19 (12–90)	30 (15–139)	−27%	−54 to 15	2.464
70th percentile of IAD	19 (12–90)	35 (17–158)	−36%	−59 to 1	2.464
80th percentile of IAD	19 (12–90)	40 (20–184)	−45%	−65 to −13	2.464
90th percentile of IAD	19 (12–90)	50 (25–226)	−56%	−72 to −30	2.464
C07AB02 (*n* = 2857)					
50th percentile of IAD	19 (12–90)	75 (45–150)	−65%	−87 to −10	6.826
60th percentile of IAD	19 (12–90)	76 (46–152)	−66%	−87 to −11	6.826
70th percentile of IAD	19 (12–90)	86 (52–172)	−70%	−88 to −21	6.826
80th percentile of IAD	19 (12–90)	99 (60–199)	−74%	−90 to −32	6.826
90th percentile of IAD	19 (12–90)	121 (74–244)	−79%	−92 to −45	6.826
C07AB02 (*n* = 894)					
50th percentile of IAD	18 (12–90)	110 (110–111)	−75%	−96 to 52	35.556
60th percentile of IAD	18 (12–90)	109 (109–110)	−74%	−96 to 54	35.556
70th percentile of IAD	18 (12–90)	126 (126–127)	−78%	−96 to 32	35.556
80th percentile of IAD	18 (12–90)	150 (150–151)	−81%	−97 to 12	35.556
90th percentile of IAD	18 (12–90)	190 (190–1920)	−85%	−98 to −12	35.556
C07AB02 (*n* = 445)					
50th percentile of IAD	18 (12–90)	173 (172–203)	−85%	−97 to −20	30.041
60th percentile of IAD	18 (12–90)	178 (177–210)	−86%	−97 to −22	30.041
70th percentile of IAD	18 (12–90)	193 (192–227)	−87%	−98 to −28	30.041
80th percentile of IAD	18 (12–90)	211 (21–249)	−88%	−98 to −35	30.041
90th percentile of IAD	18 (12–90)	241 (239–283)	−90%	−98 to −43	30.041

Abbreviations: IAD, inter‐arrival density; rWTD, reverse waiting time distribution.

## References

[1] S. Schneeweiss and J. Avorn , “A Review of Uses of Health Care Utilization Databases for Epidemiologic Research on Therapeutics,” Journal of Clinical Epidemiology 58, no. 4 (2005): 323–337. 15862718 10.1016/j.jclinepi.2004.10.012

[2] M. Hempenius , K. Luijken , A. de Boer , O. Klungel , R. Groenwold , and H. Gardarsdottir , “Quality of Reporting of Drug Exposure in Pharmacoepidemiological Studies,” Pharmacoepidemiology and Drug Safety 29, no. 9 (2020): 1141–1150. 32394589 10.1002/pds.5020 PMC7539966

[3] C. Bharat , L. Degenhardt , S. A. Pearson , et al., “A Data‐Informed Approach Using Individualised Dispensing Patterns to Estimate Medicine Exposure Periods and Dose From Pharmaceutical Claims Data,” Pharmacoepidemiology and Drug Safety 32, no. 3 (2023): 352–365. 36345837 10.1002/pds.5567 PMC10947320

[4] K. Østergaard , J. Hallas , S. Bak , R. Christensen , and D. Gaist , “Long‐Term Use of Antiplatelet Drugs by Stroke Patients: A Follow‐Up Study Based on Prescription Register Data,” European Journal of Clinical Pharmacology 68 (2012): 1631–1637. 22576729 10.1007/s00228-012-1293-7

[5] C. P. Chung , S. T. Callahan , W. O. Cooper , et al., “Individual Short‐Acting Opioids and the Risk of Opioid‐Related Adverse Events in Adolescents,” Pharmacoepidemiology and Drug Safety 28, no. 11 (2019): 1448–1456. 31418512 10.1002/pds.4872 PMC6956399

[6] A. D. Meid , D. Heider , J. B. Adler , et al., “Comparative Evaluation of Methods Approximating Drug Prescription Durations in Claims Data: Modeling, Simulation, and Application to Real Data,” Pharmacoepidemiology and Drug Safety 25, no. 12 (2016): 1434–1442. 27633276 10.1002/pds.4091

[7] A. Tanskanen , H. Taipale , M. Koponen , et al., “From Prescription Drug Purchases to Drug Use Periods–a Second Generation Method (PRE2DUP),” BMC Medical Informatics and Decision Making 15 (2015): 1–13. 25890003 10.1186/s12911-015-0140-z PMC4382934

[8] H. Støvring , A. Pottegård , and J. Hallas , “Refining Estimates of Prescription Durations by Using Observed Covariates in Pharmacoepidemiological Databases: An Application of the Reverse Waiting Time Distribution,” Pharmacoepidemiology and Drug Safety 26, no. 8 (2017): 900–908. 28466973 10.1002/pds.4216

[9] M. Meaidi , H. Støvring , K. Rostgaard , et al., “Pharmacoepidemiological Methods for Computing the Duration of Pharmacological Prescriptions Using Secondary Data Sources,” European Journal of Clinical Pharmacology 77, no. 12 (2021): 1–10. 10.1007/s00228-021-03188-9 34247270

[10] H. Støvring , A. Pottegård , and J. Hallas , “Determining Prescription Durations Based on the Parametric Waiting Time Distribution,” Pharmacoepidemiology and Drug Safety 25, no. 12 (2016): 1451–1459. 27670969 10.1002/pds.4114

[11] K. Bødkergaard , R. M. Selmer , J. Hallas , et al., “Using the Waiting Time Distribution With Random Index Dates to Estimate Prescription Durations in the Presence of Seasonal Stockpiling,” Pharmacoepidemiology and Drug Safety 29, no. 9 (2020): 1072–1078. 32436295 10.1002/pds.5026

[12] A. Pottegård and J. Hallas , “Assigning Exposure Duration to Single Prescriptions by Use of the Waiting Time Distribution,” Pharmacoepidemiology and Drug Safety 22, no. 8 (2013): 803–809. 23703741 10.1002/pds.3459

[13] M. A. Ikram , G. Brusselle , M. Ghanbari , et al., “Objectives, Design and Main Findings Until 2020 From the Rotterdam Study,” European Journal of Epidemiology 35 (2020): 483–517. 32367290 10.1007/s10654-020-00640-5 PMC7250962

[14] A. Hofman , D. Grobbee , P. De Jong , and F. Van den Ouweland , “Determinants of Disease and Disability in the Elderly: The Rotterdam Elderly Study,” European Journal of Epidemiology 7 (1991): 403–422. 1833235 10.1007/BF00145007

[15] C. Ho , N. T. Ha , D. Youens , et al., “Association Between Long‐Term Use of Calcium Channel Blockers (CCB) and the Risk of Breast Cancer: A Retrospective Longitudinal Observational Study Protocol,” BMJ Open 14, no. 3 (2024): e080982. 10.1136/bmjopen-2023-080982 PMC10928765 38458796

[16] J. Hallas , D. Gaist , and L. Bjerrum , “The Waiting Time Distribution as a Graphical Approach to Epidemiologic Measures of Drug Utilization,” Epidemiology 8, no. 6 (1997): 666–670. 9345667 10.1097/00001648-199710000-00009

[17] K. Laugesen , H. Støvring , J. Hallas , et al., “Prescription Duration and Treatment Episodes in Oral Glucocorticoid Users: Application of the Parametric Waiting Time Distribution,” Clinical Epidemiology 9 (2017): 591–600. 29180903 10.2147/CLEP.S148671 PMC5697451

[18] K. Bødkergaard , R. M. Selmer , J. Hallas , L. J. Kjerpeseth , E. Skovlund , and H. Støvring , “Using Multiple Random Index Dates With the Reverse Waiting Time Distribution Improves Precision of Estimated Prescription Durations,” Pharmacoepidemiology and Drug Safety 30, no. 12 (2021): 1727–1734. 34382713 10.1002/pds.5340

[19] H. Støvring , A. Pottegård , and J. Hallas , “Estimating Medication Stopping Fraction and Real‐Time Prevalence of Drug Use in Pharmaco‐Epidemiologic Databases. An Application of the Reverse Waiting Time Distribution,” Pharmacoepidemiology and Drug Safety 26, no. 8 (2017): 909–916. 28474439 10.1002/pds.4217

[20] L. J. Kjerpeseth , R. Selmer , I. Ariansen , Ø. Karlstad , H. Ellekjær , and E. Skovlund , “Comparative Effectiveness of Warfarin, Dabigatran, Rivaroxaban and Apixaban in Non‐Valvular Atrial Fibrillation: A Nationwide Pharmacoepidemiological Study,” PLoS One 14, no. 8 (2019): e0221500. 31449560 10.1371/journal.pone.0221500 PMC6709911

[21] A. Kemp , E. Paige , and E. Banks , Beginner's Guide to Using Pharmaceutical Benefits Scheme Data: Tips and Pitfalls (Australian National University, 2012).

[22] K. B. Nielsen and H. Stovring , WTDTTT: Stata Module to Estimate Parameters of the Ordinary and Reverse Waiting Time Distribution (WTD) by Maximum Likelihood, Statistical Software Components S458265 (Boston College Department of Economics, 2016).

[23] WHO Collaborating Centre for Drug Statistics Methodology , ATC Classification Index With DDDs Oslo, Norway 2023, https://www.whocc.no/atc_ddd_index_and_guidelines/atc_ddd_index/.

[24] J. M. Bland and D. G. Altman , “Measuring Agreement in Method Comparison Studies,” Statistical Methods in Medical Research 8, no. 2 (1999): 135–160. 10501650 10.1177/096228029900800204

[25] J. M. Thrane , H. Støvring , M. Hellfritzsch , J. Hallas , and A. Pottegård , “Empirical Validation of the Reverse Parametric Waiting Time Distribution and Standard Methods to Estimate Prescription Durations for Warfarin,” Pharmacoepidemiology and Drug Safety 27, no. 9 (2018): 1011–1018. 29952049 10.1002/pds.4581

[26] StataCorp , Stata Statistical Software: Release 18 (StataCorp LLC, 2023).

[27] H. Taipale , A. Tanskanen , M. Koponen , A.‐M. Tolppanen , J. Tiihonen , and S. Hartikainen , “Agreement Between PRE2DUP Register Data Modeling Method and Comprehensive Drug Use Interview Among Older Persons,” Clinical Epidemiology 8 (2016): 363–371. 27785101 10.2147/CLEP.S116160 PMC5066699

[28] L. Pazzagli , M. Andersen , and M. Sessa , “Pharmacological and Epidemiological Considerations While Constructing Treatment Episodes Using Observational Data: A Simulation Study,” Pharmacoepidemiology and Drug Safety 31, no. 1 (2022): 55–60. 34611960 10.1002/pds.5366

